# No effect of seed source on multiple aspects of ecosystem functioning during ecological restoration: cultivars compared to local ecotypes of dominant grasses

**DOI:** 10.1111/eva.12124

**Published:** 2013-11-12

**Authors:** Sara G Baer, David J Gibson, Danny J Gustafson, Allison M Benscoter, Lewis K Reed, Ryan E Campbell, Ryan P Klopf, Jason E Willand, Ben R Wodika

**Affiliations:** 1 Department of Plant Biology and Center for Ecology, Southern Illinois UniversityCarbondale, IL, USA; 2 Department of Biology, The CitadelCharleston, SC, USA

**Keywords:** genetic diversity, genetic structure, grassland, prairie, propagule, soil

## Abstract

Genetic principles underlie recommendations to use local seed, but a paucity of information exists on the genetic distinction and ecological consequences of using different seed sources in restorations. We established a field experiment to test whether cultivars and local ecotypes of dominant prairie grasses were genetically distinct and differentially influenced ecosystem functioning. Whole plots were assigned to cultivar and local ecotype grass sources. Three subplots within each whole plot were seeded to unique pools of subordinate species. The cultivar of the increasingly dominant grass, *Sorghastrum nutans*, was genetically different than the local ecotype, but genetic diversity was similar between the two sources. There were no differences in aboveground net primary production, soil carbon accrual, and net nitrogen mineralization rate in soil between the grass sources. Comparable productivity of the grass sources among the species pools for four years shows functional equivalence in terms of biomass production. Subordinate species comprised over half the aboveground productivity, which may have diluted the potential for documented trait differences between the grass sources to influence ecosystem processes. Regionally developed cultivars may be a suitable alternative to local ecotypes for restoration in fragmented landscapes with limited gene flow between natural and restored prairie and negligible recruitment by seed.

## Introduction

The appropriate origin (source) of plant propagules for population reintroduction is a subject of on-going debate in conservation biology and restoration ecology (Ennos et al. 1998; Hamilton 2001; Wilkinson 2001; Hufford and Mazer 2003; Rice and Emery 2003; McKay et al. 2005; Broadhurst et al. 2008; Vander Mijnsbrugge et al. 2010; Maschinski et al. 2013). The suitability of population sources for restoring degraded environments is usually unknown, so ecological restoration must rely on genetic principles (Montalvo et al. 1997; Jones 2003; Falk et al. 2006) or a ‘best guess’ of a species' adaptive potential (Broadhurst et al. 2008). In addition to reintroducing proper genetic material, ecological restoration also aims to improve the structure and function of a degraded ecosystem state. Identifying which source of genetic stock will fulfill restoration goals of reinstating biodiversity and ecosystem functioning in degraded site conditions, local climate conditions, and development of no-analog conditions represents a grand challenge that bridges the disciplines of restoration ecology and conservation biology (Harris et al. 2006; Baer 2013).

Adaptive variation within a species can arise naturally, resulting in the development of ecotypes (Turesson 1922), or through artificial selection (Darwin 1859), as in the case of plant cultivars. Local ecotypes and cultivars represent sources of genetic stock for ecological restoration. Lesica and Allendorf (1999) proposed selecting genetic stock (propagule sources) for restoration based on the size and degree of disturbance. They recommended local ecotypes (collected from natural areas with high fidelity to distance or conditions of a site) for any size restoration provided the disturbance has not been too severe, genotypic mixtures (propagules obtained from multiple populations to include potentially ‘nonlocal’ but natural sources) for large areas that are highly disturbed, and cultivars for small, severely degraded areas. Cultivars are bred for functional traits such as rapid growth rate, disease resistance, drought tolerance, and high reproductive output (e.g., seed production, viability, and germination rate) (Fehr 1987). Cultivars are generally discouraged in restoration because they may contain foreign genotypes (Hufford and Mazer 2003) and are feared aggressive, with some support for superior growth relative to wild types from two-species competition experiments (Gustafson et al. 2004a; Schroder and Presse 2013).

The genetic concerns surrounding the use of cultivars to restore native plant communities parallel those related to using commercial seed sources (Aavik et al. 2012). First, *ex situ* propagation of plants can select for genotypes best adapted to the ‘garden’ conditions of a nonlocal propagation environment (Ensslin et al. 2011). Second, harvesting seed using standardized equipment at one time can reduce phenotypic variation in only a few generations (Law and Anderson 1940). Third, commercially propagated sources, with potentially altered genetic structure, could outcross with nearby natural (remnant) populations to result in outbreeding depression and compromise fitness of local populations (Montalvo and Ellstrand 2001; Hufford and Mazer 2003). The two main mechanisms for reduced fitness of out-crossed hybrids include loss of local adaptation (Keller et al. 2000) and the disruption of co-adapted gene complexes (MaynardSmith 1998). Lastly, there is potential for cryptic invasion of a superior genotype if genetically different populations are introduced into habitats containing or adjacent to local populations (Saltonstall 2002). The debate over appropriate population sources for restoration has been fueled by the recommendation to introduce broadly sourced genotypes in restorations to promote both genetic diversity and the adaptive potential of populations to respond to environmental change (Wilkinson 2001; Rice and Emery 2003; Harris et al. 2006; Broadhurst et al. 2008). There are no studies that have simultaneously quantified the degree of genetic distinction between cultivars and local ecotype propagule sources in restored plant communities and whether these sources differentially affect ecosystem processes.

There are genetic and ecological concerns about using cultivars to restore prairie stemming from documented differences in genetic structure and plant traits that could influence ecosystem functioning. Cultivars of warm-season (C_4_) prairie grasses have been developed by the United States Department of Agriculture (USDA) to improve degraded range and agricultural lands (USDA 1995) and are a readily obtainable source of propagules for prairie restoration. There is evidence for genetic and phenotypic differences between cultivars and natural sources of prairie grasses. Gustafson et al. (2004b) demonstrated that prairie grass cultivars in restored populations contained a different genetic structure than local ecotypes in natural populations. Reduction in phenotypic variation of a cultivated prairie grass, *Andropogon gerardii*, occurred within only a few production generations (Law and Anderson 1940). Functional traits of leaf-level gas exchange (Baer et al. 2005; Lambert et al. 2011) and root production and architecture (Klopf and Baer 2011) have been shown to be enhanced in cultivars of some prairie grass species relative to ecotypes in restorations. Functional traits of dominant species largely influence grassland ecosystem functioning (Mokany et al. 2008) and dominant grasses drive the recovery of ecosystem processes during grassland restoration (Baer et al. 2002). Thus, the functional and genetic difference between cultivar and local ecotypes of prairie grasses provides a novel context to investigate whether intraspecific variation in genetic stock (seed source) differentially affects ecosystem functioning during ecological restoration.

Common garden studies are needed to evaluate the ecological consequences of using different population sources in restoration (Falk et al. 2006). We established a community common garden experiment to test whether seed sources (cultivar and local ecotype) of typically dominant prairie grasses were genetically distinct and differentially affected ecosystem functioning (energy flow and material cycling). We measured aboveground net primary productivity (ANPP), one of the most widely used indices of ecosystem functioning (Loreau et al. 2001), as well as the accrual of carbon (C) and potential net mineralization rate of nitrogen (N) in soil. We hypothesized that the grass cultivars, artificially selected for ‘improved’ traits (i.e., high growth rate and fecundity) would exhibit a different genetic structure than the local sources. We further hypothesized that cultivars would exhibit higher ANPP than the local ecotype grasses and negatively impact the ANPP of subordinate species relative to the local ecotype grass source. Lastly, we hypothesized that total ANPP would become increasingly disparate between the dominant grass seed source treatments over time because cover of these grasses tends to increase as prairie restoration proceeds (Kindscher and Tieszen 1998; Sluis 2002; Baer et al. 2003; Camill et al. 2004; Martin et al. 2005; Polley et al. 2005; Carter and Blair 2012). We anticipated that the grass cultivars would be more productive than the local ecotypes based on evidence for enhanced in leaf-level physiological processes (i.e., net photosynthesis rate, stomatal conductance, and intrinsic water use efficiency) of cultivars in this field experiment (Table 1) and enhanced root traits from a related experiment (Klopf and Baer 2011). We predicted that C accrual in soil would be higher and net N mineralization rates would be lower (greater immobilization potential) in response to greater C inputs belowground (Baer et al. 2003) in prairie restored with cultivars relative to local ecotypes of dominant grasses. The two dominant grass seed sources were sown with three unique pools of subordinate species to elucidate whether the effect of dominant species seed source on ecosystem functioning is a general phenomenon (main effect of dominant grass source across all species pools) or assemblage contingent (dominant grass source interacts with species pool), varying function under different biological conditions and associated interspecific interactions.

**Table 1:** Physiological variation between the cultivar and local ecotype seed sources of the three focal grass species (transcribed from Lambert et al. 2011). Leaf-level processes were measured four times during the 2007 growing season. The dominant grass source by date interaction (SOR × D) is explained in the footnote.

Dominant grass species	Dominant grass seed source	Net photosynthesis (*A*_net_) μmol CO_2_ m^−2^ s^−1^	Stomatal conductance (*g*_s_) mmol H_2_O m^−2^ s^−1^	Water use efficiency (WUE) μmol CO_2_ mol H_2_O^−1^
*Andropogon gerardii*	Local ecotype	15.5 ± 0.39	96.2 ± 2.02	166.1 ± 3.54
	Cultivar	20.3 ± 0.52***	120.2 ± 4.64**	175.2 ± 3.76*
*Schizachyrium scoparium*	Local ecotype	17.1 ± 0.35	120.7 ± 8.58	171.2 ± 6.92
	Cultivar	20.2 ± 0.47***	119.6 ± 4.78	189.9 ± 7.89**
*Sorghastrum nutans*	Local ecotype	21.8 ± 0.80	131.3 ± 6.14	172.9 ± 3.02
	Cultivar†	25.5 ± 0.46^SOR × D^	149.4 ± 4.66**	173.3 ± 1.85^SOR × D^

Values represent the average (± standard error) over all repeated measures and species pools.

A significant main effect of dominant grass source occurred for most processes

*** *P *<* *0.001;

** 0.001 < *P *<* *0.01;

* 0.01 < *P *<* *0.05).

† Interaction between dominant grass source and date for *A*_net_ (*P *=* *0.009) resulted from higher *A*_net_ in cultivars on the 2nd and 4th measurement dates, but there was no difference in *A*_net_ between population sources on the 1st and 3rd measurement dates. Interaction between SOR and D for WUE (*P *=* *0.015) resulted from higher WUE in the cultivar on all but the 1st measurement date.

## Methods

### Study site

We initiated a field experiment to test for hierarchical consequences of using cultivar and local ecotypes of dominant grasses on ecosystem functioning at the Southern Illinois University Agronomy Center in Carbondale, Illinois, USA (37°41′N, 89°14′W). Since 1990, climate has had a mean annual temperature of 13.4°C (average minimum and maximum of 7.4 and 19.4°C, respectively). Within the same time period, mean yearly rainfall has been 1212 mm, of which 52% has been received during the growing season, April 1 through September 30 (20-year record, Carbondale, IL) (http://weather-warehouse.com/). Total precipitation received each year of this study (2006, 2007, 2008, and 2009) was 1475, 1084, 1492, and 1545 mm, of which 664, 463, 700, and 947 mm was received during the growing season (April–September), respectively (Fig. S1). The formerly cultivated soil at the field site was classified as a fine-silty, mixed, superactive, mesic, Fragiaquic Hapludalf. The topsoil (0–0.25 m) was comprised of silt loam and the subsoil (0.25–1.30 m) of silt clay loam. Both fields used in this study were previously cultivated for maize and soybean production.

### Experimental design

**Figure 1:**
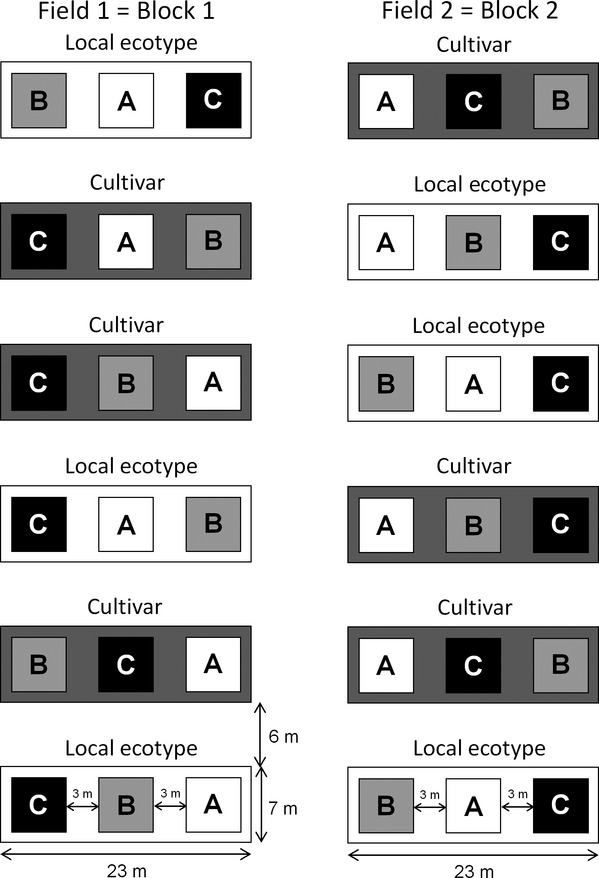
The split-plot design used to test whether dominant grass source differentially influences ecosystem functioning. Six whole plots were randomly assigned to cultivar or local ecotype seed source of three dominant grasses within two adjacent agricultural fields treated as blocks. Three unique pools (A, B, and C) of subordinate species were randomly assigned to 5 m × 5 m subplots within each whole-plot.

The field experiment consisted of a fully factorial combination of two dominant grass sources (SOR: cultivar or local ecotype) and three unique pools of subordinate native species (SPP: A = SP_A_, B = SP_B_, C = SP_C_). The experimental design was a split-plot, with whole-plots assigned to source according to a randomized complete block design (Fig. 1). Blocks were based on former agricultural field use. Each 7 m × 23 m whole plot (*n* = 12; *n* = 6 per source) contained three 5 m × 5 m subplots randomly assigned to one of three unique species pools (*n* = 36; *n* = 12 per species pool). All whole plots were separated by 6 m buffer strips.

Each whole plot was seeded with cultivar or local ecotype sources of three dominant prairie grasses: *A. gerardii* Vitman (big bluestem)*, Sorghastrum nutans* (L.) Nash (Indiangrass)*,* and *Schizachyrium scoparium* Michx. Nash (little bluestem). We used cultivars recommended by the USDA for this region based on land resource regions and plant hardiness zones (USDA 1995). The ‘Rountree’ cultivar of *A. gerardii* originated from Monona County, Iowa. This cultivar was selected at the USDA Plant Materials Center in Elsberry, Missouri and described to possess increased seedling vigor, rust resistance, forage production, seed output, and resistance to lodging. The ‘Rumsey’ cultivar of *S. nutans* originated from Jefferson County, Illinois. This cultivar was also selected at the USDA Plant Materials Center in Elsberry, Missouri and described to have increased seedling vigor, forage production, and resistance to lodging. The ‘Aldous’ cultivar of *S. scoparium* was collected from the Flint Hills region of Kansas and selected at the USDA Plant Materials Center in Manhattan, KS. At the time this project was initiated, this cultivar was the most eastern source of seed stock for this species. The ‘Aldous’ cultivar was described to have tall, vigorous, and uniform forage, good seed yield and some resistance to rust. Breeding methods for the cultivars of *A. gerardii*, *S. nutans*, and *S. scoparium* were cross-pollination, increased field selection, and ‘composite progeny of these accessions made after several generations of selection,’ respectively (USDA 1995). We hand-collected local ecotype seed of *A. gerardii*, *S. nutans*, and *S. scoparium* from four remnant prairies within 75 km of the experimental site. Remnant prairies were located 15–140 km from one another.

All whole plots were seeded with the dominant focal grasses at a rate of 300 live seeds m^−2^ (100 live seeds m^−2^ of each species). Subplots were seeded with 15 subordinate species, each sown at a rate of 20 seeds m^−2^ species^−1^ (Appendix S1). The three species pools consisted of nonoverlapping species randomly selected from a pool of 45 native species that occur in tallgrass prairie (Diboll 1997). Each species pool contained the same number of species within broadly defined functional groups (i.e., C_4_ grass, C_3_ grass, legume, and forb) (Appendix S1). Due to the extremely limited extent of native prairie in southern Illinois, seeds of subordinate species were purchased from the most local native seed supplier (Hamilton Seed Co., Hamilton, MO, USA). The origins of subordinate species were not known, but none were cultivated varieties. Any potential variation within each subordinate species was assumed to be equally distributed among subplots within a species pool. The buffer areas were sown with two native prairie grasses, *Elymus canadensis* L. and *Bouteloua curtipendula* (Michx.) Torr.

In March 2006, immediately prior to sowing, each block was disked using a tractor-mounted field cultivator. We hand-broadcasted seed of the focal dominant grasses into each whole plot and subplots were sown with each species pool. Following sowing, each whole plot was manually compacted to promote soil/seed contact. Volunteer species from the regional species pool were not removed during the 4-year study to increase relevance to restoration. The field site was burned annually in late fall or early spring when plants were dormant. Annual burning is a common management practice used to reduce undesirable weeds and promote establishment of native species in prairie restoration (Packard and Mutel 1997).

Based on counts of germinable seeds from the soil-seed bank (quantified from three 5 cm dia. ×5 cm deep soil cores removed and pooled from each whole plot, *n* = 12) and emerged seedlings (quantified from 25 cm × 25 cm × 10 cm deep soil blocks removed from each subplot, *n* = 36) in 2010 and 2011, we are confident there was little mixing of sources between whole plots during the study. In 2010, there were a total of 0, 6, and 1 germinable seeds of *A. gerardii*, *S. nutans*, and *S. scoparium* in the soil-seed bank, respectively. There were no germinable seeds of the focal grasses quantified from the soil-seed bank in 2011. There was a total of two emerged seedlings of *S. nutans* observed from all of the soil blocks in 2010 and no emerged seedlings of the three focal grasses from the soil blocks sampled in 2011 (J. Willand, unpublished data). Further, Gustafson et al. (2001) showed that adjacent plots established with different sources of *A. gerardii* contained no evidence of mixing in established plants, just in the seed. In other words, gene flow between different sources was occurring but the seeds were not establishing, so the populations remained ‘pure’ to their original source.

#### Genetic analysis of *Sorghastrum nutans*

By the second year of restoration, it was evident that *S. nutans* was becoming the dominant grass and this species was selected to evaluate genetic differences between plants that established in the cultivar and local ecotype treatments. Genomic DNA was extracted from approximately 0.5 g fresh leaf material sampled from 141 *S. nutans* plants in the cultivar (*n* = 73) and local ecotype seed source treatments (*n* = 68) using a E.Z.N.A.® plant DNA miniprep kit (Omega Bio-Tek, Norcross, GA, USA). Twenty five Inter-Simple Sequence Repeat (ISSR) primers were surveyed for the dominant grass *S. nutans*: 807 (AG)_8_T, 10 bands; 811 (GA)_8_C, 8 bands; 844 (CT)_8_RC, 8 bands; 847 (CA)_8_RC, 8 bands). The ISSR polymerase chain reaction (PCR) protocol followed that of Wolfe et al. (1998); 94°C for 1 min 30 s, 40 cycles of 94°C for 40 s, 43°C for 45 s, and 72°C for 1 min 30 s, followed by a final extension at 72°C for 5 min. The PCR profiles were visualized in 1.5% agarose gels and stained with ethidium bromide. Images were captured using a digital camera (Olympus C-4000 Zoom, Melville, NY, USA), converted to a negative image, and fragment size was estimated based on a DNA marker (#G7521; Benchtop pGEM, Promega, Madison, WI, USA). Fragment sizes were used to assign loci for each primer and bands were scored as diallelic for each locus (1 = band present, 0 = band absent).

#### Ecosystem functioning

*Aboveground Net Primary Productivity* (*ANPP*). To correspond with peak biomass, all plants were clipped from four 20 cm × 50 cm areas within a 1 m perimeter of a central 1 m^2^ within each subplot in September of each year (2006–2009). Samples were sorted into each focal grass species (*A. gerardii*, *S. nutans*, and *S. scoparium*), planted forbs, planted grasses, volunteer grasses, volunteer forbs, litter produced in that year, and litter produced in previous years. Biomass was dried at 55°C for 1 week and weighed to estimate ANPP (Briggs and Knapp 1991). Previous year's litter was not included in ANPP estimates.

*Soil C accrual*. Soil C accrual was quantified at the onset of the experiment (May 2006) and five years later (May 2011). In both years, four 2 cm dia. ×10 cm deep soil cores were removed from each subplot, separated into 0–5 and 5–10 cm depths, composited by subplot and depth, and stored at 4°C. In the laboratory, the composited samples were homogenized through a 4-mm diameter sieve. Soil C and N were determined from a subsample of soil (~30 g) dried at 55°C, ground to a fine powder, and analyzed for percent C and N on a Thermo Scientific Flash 1112 CN Analyzer distributed by CE Elantech Corporation (Lakewood, NJ, USA).

Percent C and N were converted to volumetric amounts (stocks) based on equivalent mass determined from bulk density cores. Bulk density was sampled from the 0 to 5 and 5 to 10 cm depths in 2011 using a 5.5 cm dia. intact soil coring device. One core was taken in each whole plot to characterize the bulk density of the study site. Each core was dried to a constant mass at 105°C and weighed. Bulk density was averaged by depth prior to converting C concentrations to volumetric soil mass (g m^−2^). Soil C (and N) stocks were calculated using the mass of 84.8 and 88.2 kg m^−2^ in the 0–5 and 5–10 cm, respectively.

*Potential net nitrogen mineralization rate*. Potential net N mineralization rates were determined using aerobic laboratory incubations (Robertson et al. 1999). Soil was sampled from the 0 to 5 cm soil depth in September 2009 from multiple 2 cm dia. soil cores removed and composited by subplot. Composite soil cores were sieved (4-mm) and two subsamples (~10 g) were pre-incubated in covered 125-mL Erlenmeyer flasks for 5 days at 23°C, after which half of the samples were extracted for inorganic N to serve as the initial inorganic N (N_i_) concentration. A second paired subsample was extracted for inorganic N following an incubation to serve as the final inorganic N concentration (N_f_). This process was repeated three times, resulting in three sets of incubations, each containing all treatment combinations, but different incubation times for each set (7–18 days). Soil was extracted for inorganic N by shaking each sample with 50 mL of 2 m KCl for 1 h at 200 rpm. Each extraction solution was filtered through a 0.4-μm polycarbonate membrane then frozen until analysis. Nitrate–nitrogen (nitrate + nitrite, collectively indicated as NO_3_−N) was determined by diazotization with sulfanilamide following reduction of nitrate to nitrite through a cadmium coil. Ammonium–nitrogen (NH_4_–N) was determined using the indophenol blue method. Extracts were analyzed for nitrate and ammonium on an OI Analytical Segmented Flow IV Autoanalyzer (OI Analytical Corporation, College Station, TX, USA). Daily net N mineralization rates were determined from the difference between N_f_ and N_i_ of NO_3_–N + NH_4_–N divided by the incubation period (days).

### Data analyses

We used multiresponse permutation procedure (MRPP) to test the hypothesis that there was no genetic difference between *S. nutans* plants that established in cultivar and local ecotype whole plots. MRPP is a nonparametric method of testing for group differences with the test statistic (*T*) describing the separation between groups and the within group homogeneity statistic (*A*), which indicates the effect size (McCune and Grace 2002). If *A *=* *1 all individuals within groups are identical and if *A *=* *0 heterogeneity within groups equals expectations by chance. Percent polymorphic (*PP*_ISSR_) bands and Shannon's diversity (*H′*_ISSR_) were used to characterize the genetic diversity of *S. nutans*.

All ecosystem function responses were analyzed according to a split-plot design with the whole-plot factor arranged in a randomized complete block design using the mixed model procedure in SAS (SAS version 9.1 2002). Analyses of ANPP and soil C stock included repeated measures over time. Because ANPP contained four repeated measures, we used model fit information criteria (i.e., AIC, AICC, and BIC) to select the most appropriate covariance structure (UN = unstructured, AR = autoregressive, CS = compound symmetry) for repeated measures and assigned the Kenward–Roger method to estimate degrees of freedom (Littell et al. 2006). Compound symmetry was assigned to the covariance structure for soil C and N stocks with only two repeated measures. The covariance structure (cov), numerator degrees of freedom (ndf) and denominator degrees of freedom (ddf) are reported with each F-statistic (*F*_[cov] ndf, ddf_). The least-squares means separation procedure was used if a main effect of SOR, SPP, or year (YR) occurred. If interaction between factors occurred, we used contrast and estimate statements to perform *a priori* comparisons of interest. If there was a SOR × YR interaction, we compared sources within each year and compared years within each source. If a SOR × SPP interaction occurred, we compared sources within each species pool. There were significant effects of SPP for some categories of ANPP. Significant SPP effects in the absence of any interaction with source address the role of variation in subordinate species on ecosystem functioning, which is a more general ecological rather than evolutionary application of this experiment. No three-way interactions occurred. All but one ANPP category (‘all other planted species’) were log-transformed to satisfy assumptions of normality (log *x* + 1 was used if the category contained values = 0). Net N mineralization rates were log (*x* + 1000) transformed. Significance for genetic responses was assigned at α = 0.05. Significance for the ecosystem functioning response variations was assigned at α = 0.025 rather than 0.05 due to the directional hypotheses (Zar 1984) that cultivars would be more productive, decrease the ANPP of subordinate species, increase soil C and reduce net N mineralization rates relative to prairie restored with local ecotypes.

## Results

*Sorghastrum nutans* became the most dominant grass over time, accounting for 36%, 58%, 74%, and 85% of focal grass ANPP in the 1st, 2nd, 3rd, and 4th year of restoration, respectively. There was a significant genetic difference in *S. nutans* plants growing in plots sown with the ‘Rumsey’ cultivar and *S. nutans* plants in plots sown with the local ecotype source (*T* = −1.88, *A* = 0.006, *P *<* *0.05). Genetic diversity, however, was similar between the cultivar (*PP*_ISSR_ = 0.89 ± 0.05; *H′*_ISSR_ = 3.25 ± 0.02) and local ecotype (*PP*_ISSR_ = 0.91 ± 0.05; *H′*_ISSR_ = 3.25 ± 0.07) sources of *S. nutans*.

**Figure 2:**
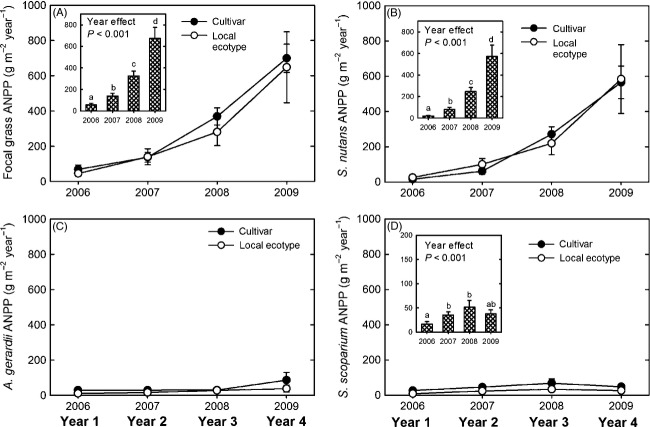
Average (± standard error) aboveground net primary productivity (ANPP) of cultivar and local ecotype sources of (A) all focal grasses, (B) *Sorghastrum nutans*, (C) *Andropogon gerardii*, and (D) *Schizachyrium scoparium* in each restoration year. Inset graphs present significant main effects of time if there was no interaction between dominant grass source and species pool. Means accompanied by the same letter were not significantly different (α = 0.025).

Collectively, the focal C_4_ grasses (*A. gerardii* + *S. nutans* + *S. scoparium*) accounted for 36%, 50%, 64%, and 61% of total ANPP of planted species over time and increased significantly each year (YR: *F*_[UN] 3, 28_ = 15.2, *P *<* *0.001) (Fig. 2A, inset graph). Focal C_4_ grass ANPP was similar between the grass sources over all species pools and years (SOR: *F*_[UN] 1, 29.2_ = 0.9, *P *=* *0.354), between sources each year (SOR × YR: *F*_[UN] 3, 28_ = 0.6, *P *=* *0.607) (Fig. 2A), and within each species pool over all years (SOR × SPP: *F*_[UN] 2, 29.2_ < 0.1, *P *=* *0.972) (Table 2).

**Table 2:** Average (± standard error) aboveground net primary productivity (g m^−2^ year^−1^) of cultivar and local ecotype sources of each focal grass species and all focal grasses in each species pool averaged over the 4 years of study.

	Species pool A	Species pool B	Species pool C
*Andropogon gerardii*			
Cultivar	124 ± 48	38 ± 8	74 ± 23
Local ecotype	133 ± 28	62 ± 28	75 ± 25
*Sorghastrum nutans*			
Cultivar	382 ± 49	427 ± 88	530 ± 74
Local ecotype	415 ± 70	449 ± 74	350 ± 110
*Schizachyrium scoparium*			
Cultivar	58 ± 16^a^	27 ± 9	40 ± 11
Local ecotype	22 ± 8^b^	18 ± 5	35 ± 13
All focal grasses			
Cultivar	564 ± 28	492 ± 101	644 ± 101
Local ecotype	571 ± 66	529 ± 59	460 ± 113

Source means within a species pool accompanied by different letters were significantly different (α = 0.025).

The three focal grass species (*S. nutans*, *A. gerardii*, and *S. scoparium*) exhibited different patterns in ANPP over time, but no difference between seed sources each year, with the exception of *S. scoparium* that exhibited an interaction between species pool and dominant grass seed source. *Sorghastrum nutans* was the only focal grass to increase in ANPP each year (YR: *F*_[UN] 3, 28_ = 16.9, *P *<* *0.001) (Fig. 2B, inset graph) and the ANPP of *S. nutans* was similar between sources each year (SOR × YR: *F*_[UN]3, 28_ = 3.89, *P *=* *0.926) and within each species pool over all years (SOR × SPP: *F*_[UN]2, 27.8_ = 0.16, *P *=* *0.856) (Table 2). Likewise, there was no difference in ANPP between cultivar and local ecotype of *A. gerardii* over all years (SOR main effect: *F*_[AR] 2, 8.7_ = 0.9, *P *=* *0.377), in any year (SOR × YR: *F*_[AR] 3, 74_ = 0.87, *P *=* *0.426) (Fig. 2C), or within each species pool (SOR × SPP: *F*_[AR]2, 28.3_ = 0.01, *P *=* *0.991) (Table 2). *Schizachyrium scoparium* ANPP varied among years (YR: *F*_[AR] 3, 81.4_ = 6.2, *P *=* *0.001) (Fig. 2D) and was the only focal grass to exhibit an interaction (but weak, *P* > 0.025) between source and species pool (SOR × SPP: *F*_[AR] 2, 29.2_ = 3.6, *P *=* *0.039). This interaction resulted from higher ANPP in the cultivar source of *S. scoparium* in SP_A_, but not the other species pools (Table 2). This source effect was not reflected in focal C_4_ grass, total, or planted ANPP due to the overall low productivity of this species.

**Figure 3:**
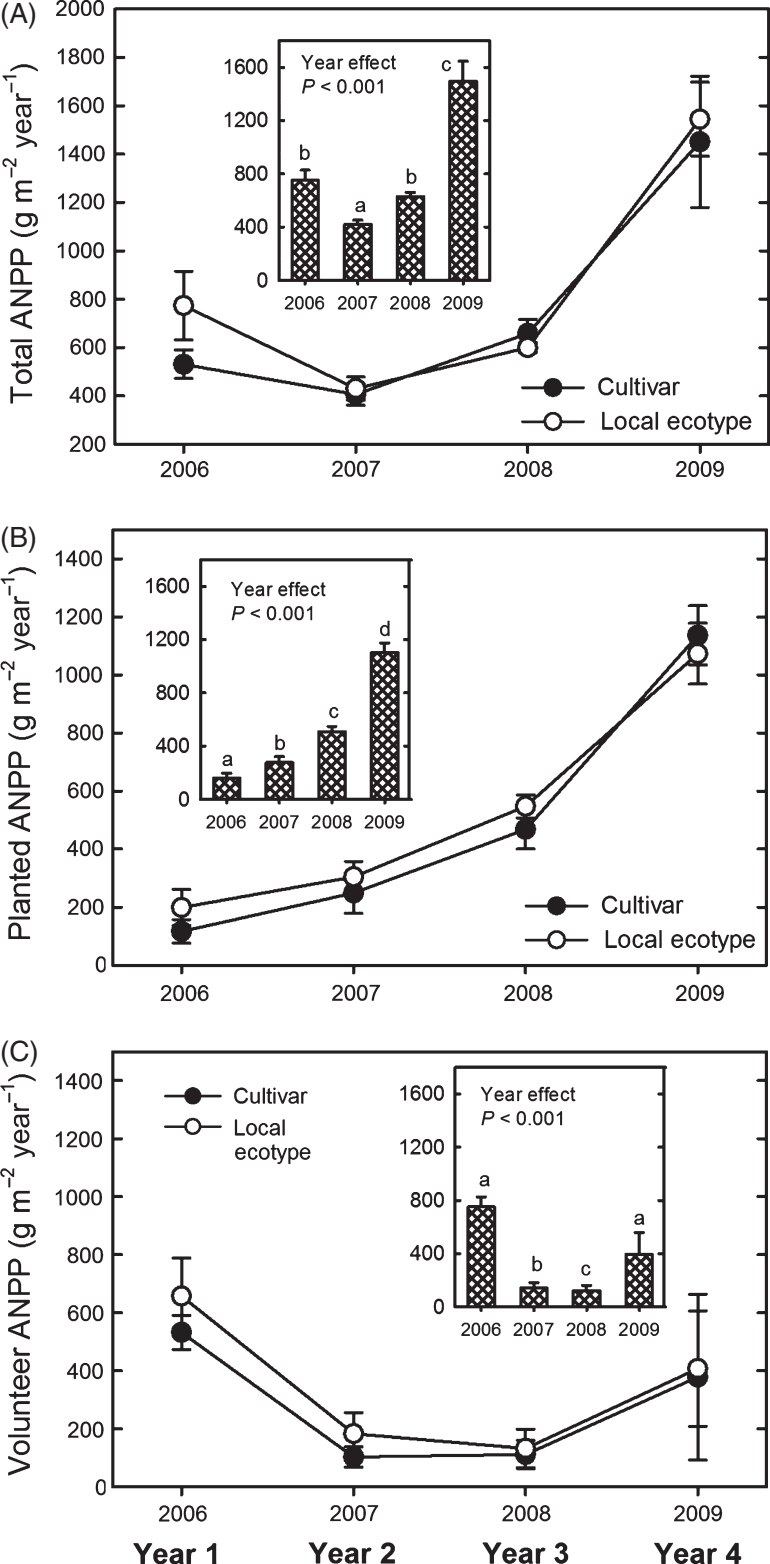
Average (± standard error) aboveground net primary productivity of (A) all species, (B) planted species, and (C) volunteer species each year in the cultivar and local ecotype dominant grass treatments. Inset graphs present significant main effects of time if there was no interaction between dominant grass source and species pool. Means accompanied by the same letter were not significantly different (α = 0.025).

Total, planted, and volunteer ANPP showed similar responses to dominant grass source. Total ANPP changed significantly over time (YR: *F*_[CS] 3,90_ = 36.9, *P *<* *0.001) but was not affected by dominant grass source in any year (SOR × YR: *F*_[CS] 2, 20_ = 1.0, *P *=* *0.397) or over all years (SOR: *F*_[CS] 1, 10_ < 0.1, *P *=* *0.814) (Fig. 3A). Planted ANPP increased each year (YR: *F*_[UN] 3, 28_ = 31.4, *P *<* *0.001) (Fig. 3B, inset graph), accounting for 17%, 49%, 70%, and 70% of total ANPP over time. The ANPP of planted species was similar between the dominant grass sources over all years (SOR: *F*_[UN] 1, 16.9_ = 2.0, *P *=* *0.171) and within each year (SOR × YR: *F*_[UN] 3, 28_ = 0.2, *P *=* *0.869) (Fig. 3B). The ANPP of volunteer species varied with time (YR: *F*_[UN] 3, 28_ = 58.3, *P *<* *0.001) (Fig. 3C, inset graph) but was not affected by dominant grass source over all years or in any year (SOR: F_[UN] 1, 20.1_ = 0.7, *P *=* *0.413; SOR × YR: *F*_[UN] 3, 28_ = 0.8, *P *=* *0.534) (Fig. 3C). Volunteer ANPP was highest in year 1 and the decline in ANPP of this group in years 2 and 3 was due to the loss of annual weedy species. The increase in volunteer ANPP in year 4 was attributed to the colonization of *Solidago canadensis* L. (Gibson et al. 2013).

**Figure 4:**
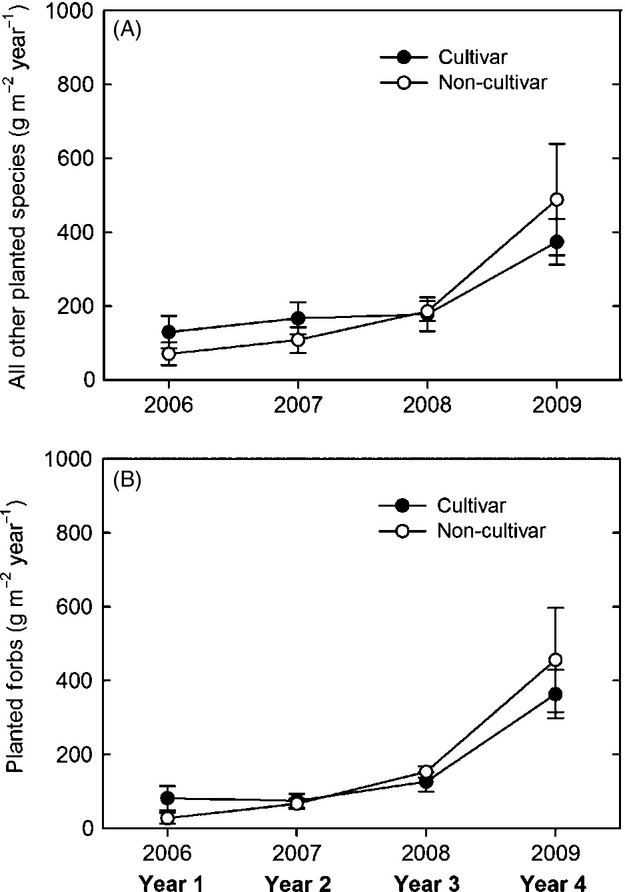
Average (± standard error) aboveground net primary productivity of (A) planted species excluding the dominant grasses and (B) all planted forbs each year in the cultivar and local ecotype dominant grass treatments.

Subordinate species (planted and volunteer combined) comprised 93%, 75%, 53%, and 57% of the total ANPP corresponding to the 1st through the 4th year of community establishment. To elucidate whether dominant grass source differentially affected the ANPP of restored subordinate species, we examined the ANPP of all planted species *excluding the focal grasses* by two refined classes: all other planted species and planted forbs. The ANPP of all other planted species was not affected by dominant grass source over all years (SOR: *F*_[UN] 1, 28.8_ < 0.1, *P *=* *0.973) or within any year (SOR × YR: *F*_[UN] 3, 28_ = 0.5, *P *=* *0.720) (Fig. 4A). Planted forb ANPP was also similar among the dominant grass sources over all years and within each year (SOR: *F*_[CS] 1, 29_ = 1.9, *P *=* *0.185; SOR × YR: *F*_[CS] 3, 90_ = 1.7, *P *=* *0.167) (Fig. 4B).

Belowground measures of ecosystem function were similar in prairie established with the cultivars and local ecotypes, showing no interaction between dominant grass source and time or dominant grass source and species pool. Soil C stock increased over time across all treatments from 1831 ± 30 g m^−2^ in 2006 to 2183 ± 36 g m^−2^ in 2011 (YR: *F*_[CS] 1, 31_ = 75.1, *P *<* *0.001). Total soil C stocks increased to the same extent in prairie restored with the cultivar and local ecotype grass sources and did not interact with species pool (SOR × SPP: *F*_[CS] 2, 20_ = 0.13, *P *= 0.880). As a result, soil C stocks were similar in prairie established for 5 years with each source of grasses over all species pools (cultivar: 2182 ± 70 g m^−2^; local ecotype: 2183 ± 30 g m^−2^). Total soil N increased over time at the same rate in prairie restored with both focal grass sources and across all species pools. Total soil N stock increased from 219.6 ± 2.64 g m^−2^ in 2006 to 234.6 ± 4.2 g m^−2^ in 2011 (Year main effect: F_[CS]1,30_ = 12.3, *P *=* *0.001). There was no effect of source (*F*_1, 9_ = 0.13, *P *=* *0.732) or interaction between source and species pool (*F*_2, 20_ = 0.93, *P *=* *0.409) on potential net N mineralization rate. Across all species pools, there was net immobilization of N in both the cultivar (−96.9 ± 41.2 μg g^−1^ day^−1^) and local ecotype (−36.2 ± 98.7 μg g^−1^ day^−1^) restored soil; the large variability in potential net N mineralization rates was due to the species pools.

## Discussion

Genetic differences between restored and natural populations are expected if the source of a restored population contains a different genetic history (Honjo et al. 2008), which could be shaped by geographic distance and associated variation in environmental conditions, or selection for traits during commercial production. Differences between natural and artificial (commercial) seed sources have been detected for a number of species in restorations, including a perennial dune grass (*Ammophila breviligulata* Fern.; Fant et al. 2008), a rare endemic tree species (*Metasequoia glyptostroboides*; Li et al. 2005), as well as the grasses studied in this experiment (Gustafson et al. 1999, 2004b). Aavik et al. (2012) documented similar genetic diversity among natural and restored populations of a wetland plant (*Lychnis flos-cuculi*), but there was a difference in genetic structure and higher inbreeding depression in the restored populations despite measures taken to avoid genetic change in the propagation process. Alternatively, the genetic structure of restored populations can group within that of natural populations when natural populations serve as the source of propagules for restoration (McGlaughlin et al. 2002).

*Sorghastrum nutans* became the most dominant grass species in this restoration experiment and genetic analyses indicated a difference in the genetic structure between prairie established with cultivar and locally collected seed, but no difference in genetic diversity between the two sources of this dominant species. This agrees with Gustafson et al.'s (2004b) study of natural and cultivar-restored prairies in this region, which demonstrated most genetic diversity in *S. nutans* (as well as *A. gerardii*) was retained within rather than between populations. This might be expected for a wind-pollinated outcrossing species. The difference in genetic structure was not surprising given the natural and commercial sources of the populations and confirms the efficacy of the dominant grass seed source treatments in this experiment. The low effect size could be due to the close proximity (within 100 km) of the remnant population seed sources (Jackson Co., Murphysboro, IL, USA) to the cultivar origin (Jefferson Co., Mount Vernon, IL, USA). The similarity in genetic diversity between the commercial source and natural populations was unexpected because whole plots assigned to the local ecotype grass source were established using seed collected from multiple remnant populations representing very different habitats (i.e., deep soil lowland prairie, shallow soil glade, and loess hill prairie) and presumably limited gene flow among populations in the agricultural and forested landscape of this region. It is possible that pre-adaptation to local conditions (site effects) selected or served as a filter for the most suitable genotypes (Gibson et al. 2012). Prairie grass cultivars are selected for traits in agricultural environments at USDA Plant Materials Centers (USDA 1995). The agricultural soil conditions at the onset of the restoration may have contributed to a site effect and the low effect size of the difference in genetic structure between the cultivar and natural population sources of *S. nutans*. These results inform the debate about genetic diversity of cultivars generally but are more relevant to demonstrating that a different genetic structure could result in prairie restored with cultivars. We have limited understanding of whether cultivars differ from local ecotypes in adaptive variation.

Intraspecific variation in plant traits can cause divergence in ecosystem processes (Whitham et al. 2003; Crutsinger et al. 2006; Orwin et al. 2010; Cook-Patton et al. 2011). We expected cultivar and local ecotype sources of prairie grasses would differentially affect ecosystem functioning, either through differences in ANPP between the dominant grass sources or the dominant grass sources differentially affecting the ANPP of other species, for example, biological filtering during community assembly (Gibson et al. 2012). Despite previously documented trait variation in the focal grasses (Klopf and Baer 2011; Lambert et al. 2011), prairie restored with cultivar and local ecotype sources of grasses did not differentially affect multiple measures of ecosystem functioning (i.e., ANPP, C accrual in soil, and potential net N mineralization rates). Further, similarity in ANPP between the two seed sources occurred over 4 years with substantial variation in monthly rainfall (Fig. S1) and across multiple pools of subordinate species, underscoring the functional similarity of these sources under temporally varying abiotic conditions and contrasting interspecific interactions, respectively. These results imply similar plasticity, at least in terms of biomass production, between the cultivar and local sources. However, numerous other aspects of potential functional variation between cultivar and local ecotypes in restored communities remain unknown (i.e., disease resistance, drought tolerance, quality and timing of resource provision to consumers, and long-term survival).

There are several possible reasons why the documented variation in functional traits between source populations were not reflected in ecosystem functioning. First, trait variation between the cultivar and local grass sources may not have been disparate enough to differentially influence ecosystem functioning. Second, the focal species that exhibited the greatest variation in leaf-level physiological traits between the two seed sources in this experiment (*A. gerardii*) did not become dominant and the increasingly dominant species (*S. nutans*) exhibited the least difference in these traits between the two seed sources (Lambert et al. 2011). Third, the origin of the *S. nutans* cultivar was closest to the remnant populations and restoration site relative to *A. gerardii* and *S. scoparium*, overriding genetic dissimilarity. Interpretation of results from this study may be contingent upon the relative success of each focal grass species, but they reflect the common phenomenon of stochastic species establishment in ecological restoration (Zedler 2000; Trowbridge 2007).

Finally, our results may not conform to demonstrated trait variation differentially influencing ecosystem processes due to the community context of our experiment, which included interspecific plant interactions. Studies that have linked intraspecific variation to ecosystem processes have been conducted using monoculture stands of a focal species from different origins (Seliskar et al. 2002; Orwin et al. 2010) or measured ecosystem processes directly influenced by the focal species, for example, decomposition rate of litter produced by distinct genotypes that vary in tissue biochemistry (Driebe and Whitham 2000; Madritch and Hunter 2003; Schweitzer et al. 2004). We introduced multiple grass species and a suite of subordinate species to increase the relevance of this study to restoration and the goal of restoring biodiversity (Rey Benayas et al. 2009; Brudvig 2011). Subordinate species (planted and volunteers combined) comprised more than half of the total ANPP in all years of this experiment. Further, we have documented negligible effects of C_4_ grass source in this study on plant community composition, species richness, and diversity (Gibson et al. 2013). Similar productivity and composition within each species pool seeded with the cultivar and local ecotype sources of grasses contributed to similar functioning. We propose that the potential for intraspecific trait variation in dominant species to regulate ecosystem functioning may be diluted if there is high productivity of subordinate species, which suggests overriding interspecific interactions. This supposition is corroborated by a recent finding that ‘interspecific indirect genetic effects’ (variation in plant traits of a focal species resulting from genetic variation in neighbors) had a stronger influence on belowground traits (biomass) than genotypic variation of focal species (Genung et al. 2013).

### Implications for ecological restoration

Restoration guidelines generally recommend the use of local seed for ecological and genetic reasons, but there is not always empirical support for the principle-based rationale. Locally sourced plants are presumed to establish better, provision resources to higher trophic levels at the most appropriate time, restore mutualistic species interactions, and minimize evolutionary and ecological risk, that is, genetic pollution from introduction of maladapted genotypes (Linhart and Grant 1996; Broadhurst et al. 2008). However, local ecotypes do not always outperform nonlocal sources (Bischoff et al. 2010) and crossing multiple population sources does not always result in outbreeding depression (Edmands 2007). Broadhurst et al. (2008) suggest that using local populations may not always be ‘best’ for restoration, particularly when natural (wild) populations contain a limited supply of propagules and corresponding lower genetic diversity due to habitat loss and fragmentation. This circumstance describes the region where this study was conducted and many states in the US Midwest, where <1% of the historic extent of tallgrass prairie remains (Samson and Knopf 1994).

In contrast to the concern that nonlocal sources could be poorly adapted to a restoration site relative to local sources (Montalvo and Ellstrand 2000; Wilkinson 2001; Bischoff et al. 2006; Broadhurst et al. 2008; Vander Mijnsbrugge et al. 2010), this study documents similarity in genetic diversity and functioning of communities restored with cultivars and local ecotypes. Cultivars are generally not considered locally sourced, although many regionally specific prairie grass cultivars have been developed, based mostly on cold hardiness and drought resistance (USDA 1995). Few studies have examined the extent to which these cultivars are genetically differentiated from natural populations (Gustafson et al. 1999, 2004b). The origin of cultivar genetic stock can, in some instances, be regional and indistinguishable from natural populations at fairly large geographic scales. For example, Casler et al. (2007) investigated genetic diversity of 46 natural populations and 11 cultivars of switchgrass (*Panicum virgatum*) and found no detectable differences in genetic structure of cultivars and natural populations in the northern and central US, but a small amount of variation among populations was explained by hardiness zones and ecoregions. At a more local spatial scale, Gustafson et al. (2004a) documented differences in the genetic structure of cultivar and local ecotypes of *A. gerardii* but observed higher genetic diversity within than between remnant prairie and prairie restored with local or cultivar seed sources of *A. gerardii* and *S. nutans*. There is indication that development of prairie grass cultivars can retain genetic diversity (Gustafson et al. 2004b; Casler et al. 2007). The high genetic variation within both population sources and close proximity of the cultivar origin and local ecotype population sources of *S. nutans* in this study likely contributed to similar aboveground functioning in prairie restored with each source.

Tallgrass prairie restoration practitioners generally prefer to use locally sourced seed (Rowe 2010) and cultivars are often discouraged. There is a perception that cultivars of native grasses will be more aggressive and successful than local ecotypes in restorations because they have been selected for traits to improve vigor (Fehr 1987). Evidence of enhanced growth-related traits of prairie grass cultivars relative to local ecotype sources provide empirical rationale for the anticipated success of these cultivars in restorations (Gustafson et al. 2004a; Klopf and Baer 2011; Lambert et al. 2011). But consistent with this study, Wilsey (2010) found no differences in the productivity of cultivar and noncultivar sources of C_4_ grasses in low-diversity grassland restorations. Multiple studies are needed to elucidate whether different population sources have consequences for aboveground functioning due to potential site effects on productivity. These corroborative results fuel an uncertainty about the general dogma that discourages using cultivars in restorations. However, the potential for genetic pollution (Hufford and Mazer 2003) and ‘extended phenotype’ effects on associated herbivore and decomposer communities (Schweitzer et al. 2004; Bailey et al. 2006) supports using cultivars primarily where no or few remaining remnant populations persist.

Grassland restored with either nonlocal, cultivar, or local ecotype sources in highly fragmented landscapes may face similar concerns with respect to adaptive potential. Fant et al. (2008) showed that a restored dune grass population persisted with different genetic structure, possibly due to insufficient gene flow with natural populations or seedling recruitment. There is also limited recruitment of new individuals by seed in this experiment (J. Willand, unpublished data), as well as prairie restorations conducted at larger scales using local ecotypes (Willand et al. 2013), and in remnant tallgrass prairie (Benson and Hartnett 2006). Thus, a distinct genetic structure could persist for a very long time in isolated populations of out-crossing perennial species where there is negligible recruitment by seed (Gustafson et al. 2001).

In closing, ecological restoration offers a novel context to test hypotheses in ecology and evolutionary biology (reviewed by Baer 2013). We examined whether seed sources of dominant species differentially influence ecosystem functioning, directly through variation in dominant species' productivity or indirectly through dominant species' effect on the productivity of subordinate species. We found no strong effects of dominant grass source on above-or belowground ecosystem functioning. This study informs a controversial issue in conservation regarding the ecological consequences of population sources for reintroduction. These results contribute to a developing body of knowledge on the applicability of genetic principles to the restoration of plant communities and ecosystem functioning.

## Data archiving statement

Raw experimental data for this manuscript are available from the Dryad Digital Repository: doi:10.5061/dryad.c8r43.

## Supporting Information

**Appendix S1**. Composition of each species pool. **Figure S1**. Total precipitation received each month at the experiment site for the duration of study (2006–2009).
