# Supplementary material for: No effect of seed source on multiple aspects of ecosystem functioning during ecological restoration: cultivars compared to local ecotypes of dominant grasses
Source: Evol Appl. 2013 Nov 12;7(2):323–35. doi: 10.1111/eva.12124 (PMC3927892; doi:10.1111/eva.12124)
Supplement: Supplementary file 1 — Appendix S1. Composition of each species pool. Figure S1. Total precipitation received each month at the experiment site for the duration of study (2006–2009). [file eva0007-0323-sd1.docx]

**Appendix 1: Composition of each species pool.** Species and functional group designation of each species in the three unique species pools seeded with dominant grass source. Nomenclature follows the United States Department of Agriculture PLANTS Database (http://plants.usda.gov).

| \| Functional Group \| Species  Pool  A \| Species  Pool  B \| Species  Pool  C \| \| --- \| --- \| --- \| --- \| \|  \|  \|  \|  \| \|  \|  \|  \|  \| \| Non-Legume Forb \| *Asclepias tuberosa* \| *Achillea millefolium* \| *Symphyotrichum oolentangiense* \| \| Non-Legume Forb \| *Asclepias verticillata* \| *Asclepias syriaca* \| *Heliopsis helianthoides* \| \| Non-Legume Forb \| *Symphyotrichum oblongifolium* \| *Echinacea purpurea* \| *Brickella eupatorioides* \| \| Non-Legume Forb \| *Callirhoe involucrata* \| *Eupatorium altissimum* \| *Monarda fistulosa* \| \| Non-Legume Forb \| *Delphinium carolinianum* \| *Liatris pycnostachya* \| *Penstemon digitalis* \| \| Non-Legume Forb \| *Oenothera macrocarpa* \| *Oenothera biennis* \| *Rudbeckia hirta* \| \| Non-Legume Forb \| *Ratibida pinnata* \| *Ruellia humilis* \| *Silphium laciniatum* \| \| Non-Legume Forb \| *Rosa arkansana* \| *Silphium integrifolium* \| *Solidago speciosa* \| \| Non-Legume Forb \| *Packera plattensis* \| *Oligoneuron rigidum* \| *Vernonia fasciculata* \| \| Legume \| *Baptisia alba* \| *Amorpha canescens* \| *Astragalus canadensis* \| \| Legume \| *Chamaecrista fasciculata* \| *Dalea candida* \| *Baptisia bracteata* \| \| Legume \| *Mimosa microphylla* \| *Desmanthus illinoensis* \| *Lespedeza capitata* \| \| Legume \| *Psoralidium tenuiflorum* \| *Desmodium illinoense* \| *Dalea purpurea* \| \| C3 Grass \| *Elymus canadensis* \| *Koeleria macrantha* \| *Agrostis hyemalis* \| \| C4 Grass \| *Sporobolus heterolepis* \| *Bouteloua curtipendula* \| *Panicum virgatum* \| \|  \|  \|  \|  \| \|  \|  \|  \|  \| |  |  |
| --- | --- | --- | --- | --- | --- | --- | --- | --- | --- | --- | --- | --- | --- | --- | --- | --- | --- | --- | --- | --- | --- | --- | --- | --- | --- | --- | --- | --- | --- | --- | --- | --- | --- | --- | --- | --- | --- | --- | --- | --- | --- | --- | --- | --- | --- | --- | --- | --- | --- | --- | --- | --- | --- | --- | --- | --- | --- | --- | --- | --- | --- | --- | --- | --- | --- | --- | --- | --- | --- | --- | --- | --- | --- | --- | --- | --- | --- | --- | --- | --- | --- | --- |

**Supplemental Information.** Total precipitation received each month at the experiment site for the duration of study (2006-2009). The growing season for tallgrass prairie typically extends from April through September. Daily rainfall data was obtained from a NOAA weather station located <1 km from the study site (latitude: 37°43, longitude: −89°09).
